# Inter- and intrahemispheric sources of vestibular signals to V1

**DOI:** 10.1073/pnas.2503181122

**Published:** 2025-10-10

**Authors:** Guy Bouvier, Alessandro Sanzeni, Elizabeth Hamada, Nicolas Brunel, Massimo Scanziani

**Affiliations:** ^a^Department of Physiology, University of California San Francisco, San Francisco, CA 94158; ^b^HHMI, University of California San Francisco, San Francisco, CA 94158; ^c^CNRS, Institut des Neurosciences Paris-Saclay, Université Paris-Saclay, Saclay 91400, France; ^d^Department of Computing Sciences, Bocconi University, Milan 20100, Italy; ^e^Center for Theoretical Neuroscience, Columbia University, New York, NY 10027; ^f^Mortimer B Zuckerman Mind Brain Behavior Institute, Columbia University, New York, NY 10027; ^g^Department of Neurobiology, Duke University, Durham, NC 27710; ^h^Department of Neurology, University of California San Francisco, San Francisco, CA 94158

**Keywords:** visual cortex, thalamus, vestibular system, mouse

## Abstract

Head movements are sensed by the vestibular organs. Unlike classical senses, signals from vestibular organs are not selectively conveyed to a dedicated cortical area but are broadcast throughout the cortex. This distributed processing pattern reflects the fundamental role of vestibular information in contextual modulation across diverse cortical computations. Surprisingly, the routes taken by vestibular signals to reach the cortex are still largely uncharted. Here, we show that the primary visual cortex (V1) receives real-time head movement signals—direction, velocity, and acceleration—from the ipsilateral pulvinar and contralateral visual cortex (VC). The ipsilateral pulvinar provides the main head movement signal, with a bias toward contraversive movements (e.g., clockwise movements in left V1). Conversely, the contralateral VC provides head movement signals during ipsiversive movements. Crucially, head movement variables encoded in V1 are already encoded in the pulvinar, suggesting that those variables are computed subcortically. Thus, the convergence of inter- and intrahemispheric signals endows V1 with a rich representation of the animal’s head movements.

## Significance

Information about head motion is fundamental to the visual interpretation of our environment. Indeed, head motion signals originating from the vestibular system robustly modulate activity in the visual cortex (VC). Despite this profound modulation, however, we still do not know how these signals reach the VC. Furthermore, we have only a very rudimentary understanding of what aspects of head motion are transmitted. We found that two distinct pathways deliver head motion signals to the primary VC: the pulvinar nucleus of the thalamus and the contralateral VC. These pathways provide complementary directional information about head movements, revealing how the brain integrates movement-related signals within visual circuits that might help maintaining stable vision during motion.

Many of the sensory organs through which we perceive the world are located in our head, for example, the eyes. To accurately represent our surroundings, sensory systems in the brain must combine their primary source of sensory information, e.g., visual signals, with information about head movements in space (1, 2). The vestibular organs, located in the inner ear, provide this information by transforming head movements into neural signals. Unlike other senses, however, these head movement signals are not selectively processed by a dedicated cortical area, but are instead broadcast throughout the brain (3–9). Previous studies in rodents have demonstrated that neurons in primary sensory areas such as the primary visual cortex (V1) robustly respond to head movements, even in the absence of visual stimuli (6–8). These responses depend on vestibular organs and dynamically track the time course of head movements, demonstrating specificity for aspects such as direction and velocity (7, 8). In contrast to our thorough understanding of the origin and processing of visual signals in V1, our understanding of head movement signals in this structure is still rudimentary. Is there a laminar organization in the representation of head movement signals in V1 as there is for visual information? Are head movement variables, such as direction and speed, computed in V1 or inherited from upstream structures? And, crucially, what are these upstream structures that relay head movement information to V1?

Here, we use the mouse as a model system to determine the dynamics of V1 activity in response to head movement and reveal that the pulvinar nucleus of the thalamus, which receives axonal projections from the deep cerebellar nuclei (DCN), represents the main source of head movement signals to V1. We show that head movement variables, like direction and speed, are more accurately represented in the deep than the superficial layers of V1 and that, rather than being computed ex novo in V1, these variables are inherited from the pulvinar—also known as the lateral posterior nucleus in rodents. The pulvinar, however, provides V1 with head movement signals that are biased toward contraversive movements [e.g., clockwise (CW) movements in left V1]. Unexpectedly, we show that the contralateral VC also provides V1 with head movement signals which, in contrast to the pulvinar, are stronger during ipsiversive head movements, and thus counterbalance the pulvinar bias. These results show that V1’s rich representation of an animal’s head movement variables results from the integration of inter- and intrahemispheric signals.

## Results

### Encoding of Head Movements in V1.

**Fig. 1.**
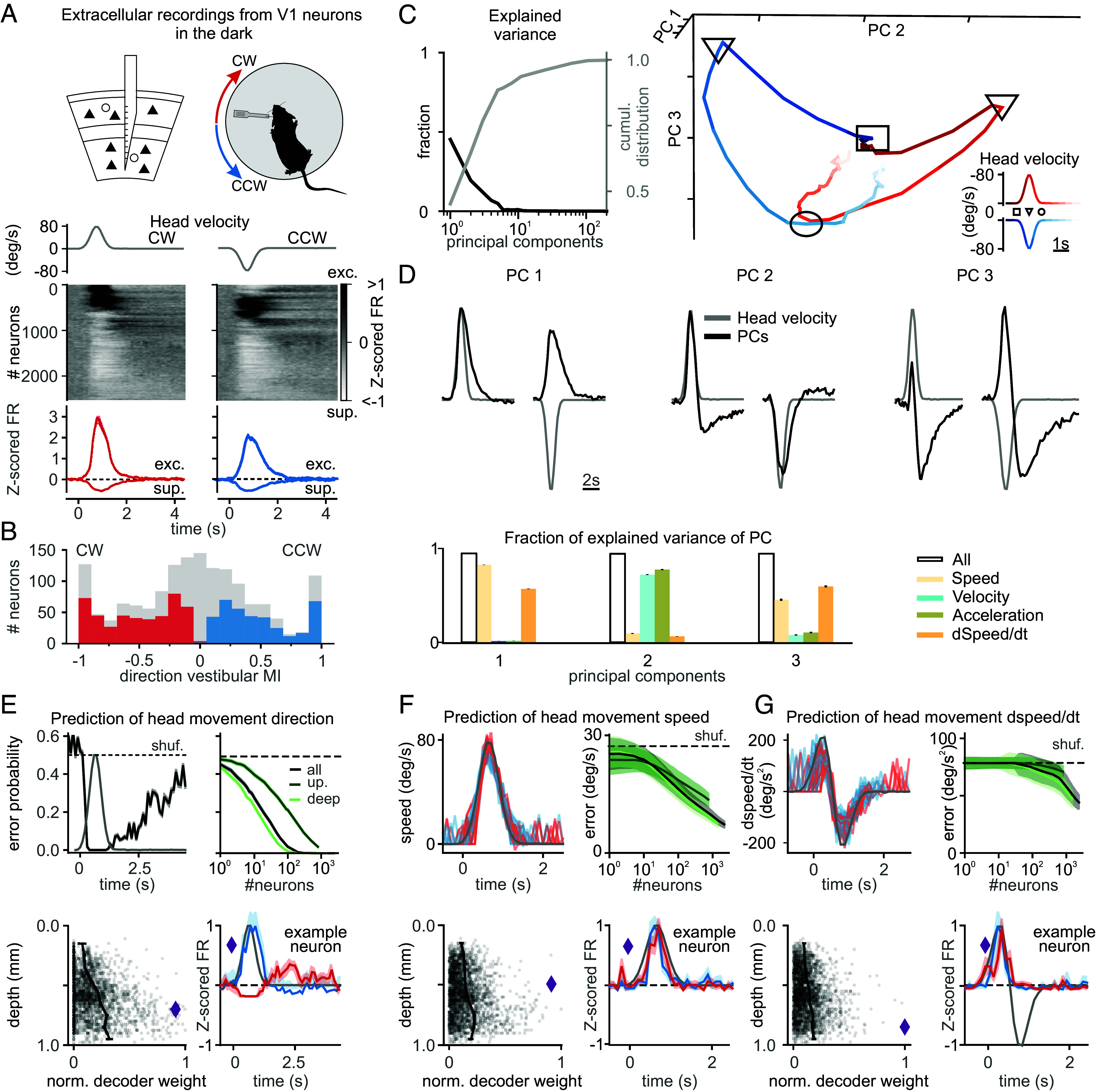
V1 encodes head movements in the absence of visual stimuli. (*A*, *Top*) Experimental configuration. A linear probe spanning all cortical layers was inserted in the left V1 of a head-fixed, awake mouse to record the response to CW and CCW rotations of a table in the dark. (*Middle*) Uniform manifold approximation and projection (UMAP) sorting of the responses, represented by the averaged Z-score of the FR across neurons. Neurons were ordered using UMAP with cross-validation. (*Bottom*) Average Z-scored FR of neurons significantly excited (exc.) or suppressed (sup.) by head movement in CW (red) and CCW (blue) direction. The gray traces on top are the velocity profile. (*B*) Distribution of vestibular modulation indices (vMI) for neurons significantly modulated during head rotations. direction vMI = (CCW rotation FR − CW stationary FR)/(CCW rotation FR + CW stationary FR)). Positive and negative values of direction vMI represent neurons preferring CCW and CW turns, respectively. The histogram shows the direction vMI of those neurons whose FR is significantly modulated by head movement: Prefers CW rotations (red), Prefers CCW rotations (blue), Significantly modulated by head movements without direction preference (gray; *Materials and Methods*). (*C*) Principal component (PC) analysis of V1 population activity during head movements. (*Left*) Fraction of explained variance (black) and its cumulative distribution (gray) as a function of increasing PC. (*Right*) Three-dimensional representation of the first three PCs. Note the separation of CW (red) and CCW (blue) representations (*Inset*: time is represented as progressing from dark to light; the square, triangle, and circle symbols indicate beginning, peak, and end of the rotation, respectively). (*D*, *Top*) Population dynamics along the first three PCs (black) superimposed on the velocity profile (gray). (*Bottom*) Variance explained in each PC by models including speed, velocity, and their time derivatives either all together (empty bar) or separately (color). (*E*) Representation of head movement direction in a trial. (*Top Left*) Decoding error probability as a function of time. (*Top Right*) Decoding error probability as a function of the number of neurons (computed at peak velocity). Light and dark green indicate deep and upper layers, respectively. (*Bottom Left*) Decoder weight of individual neurons (gray dots) and population average (black connected dots; bin = 100 μm) as a function of depth. (*Bottom Right*) Example neuron (purple diamond) selected using the highest decoder weight for head movement direction, at the peak, and 2 s after the peak velocity (red: CW; blue: CCW). (*F*) Prediction of head angular speed. (*Top Left*) Decoding head angular speed as a function of time. (*Top Right*) Error as a function of number of neurons (color indicates depth as in *E*). (*Bottom Left*) Decoder weight of single neuron as a function of depth. (*Bottom Right*) Example neuron (purple diamond) selected using highest decoder weight for head angular speed decoding (red: CW; blue: CCW). (*G*) Analogous to *F* but for the time derivative of speed.

We recorded extracellular activity in the left V1 of head-fixed, awake mice in response to vestibular stimulation, delivered in the dark, by rotating the animal along the horizontal plane with a servo-controlled rotating table (Fig. 1 *A*, *Top*). This protocol elicits responses in V1 that entirely depend on the vestibular organs (7, 8). Most V1 neurons (63%; 1,490/2,355 neurons; N = 37 mice) responded to CW; (i.e., contraversive relative to left V1: 49%; 1,152/2,355 neurons) and/or counterclockwise (CCW; i.e., ipsiversive relative to left V1: 47%; 1,112/2,355 neurons) rotations of the table, by either increasing or decreasing their firing rate (FR), as described previously (see *Materials and Methods* for class assignment criteria and statistical tests throughout).

Head movements trigger compensatory eye movements in the opposite direction via the vestibulo-ocular reflex (VOR). Since eye movements are known to modulate V1 neuronal activity (10, 11), we tested whether V1 neurons can respond to head movements independently of eye movements. For this, we implemented a vestibular stimulation protocol that eliminates VOR, called VOR cancellation (*Materials and Methods*). Here, instead of rotating the animal in the dark, we displayed a vertical grating on a virtual drum that rotates along the horizontal plane together with the animal, thereby preventing compensatory eye movements (*SI Appendix*, Fig. S1). Even during VOR cancellation, 66% of V1 neurons responded to head movements (231/351 neurons; N = 5 mice; *P* = 0.18 compared to vestibular stimulation in the dark). Thus, the activity of V1 neurons is strongly modulated by head movements even in the absence of eye movements. All subsequent experiments were conducted in the dark.

Among left V1 neurons that responded to CW and/or CCW rotations, changes in FR were, on average, larger for CW than CCW rotations (|Z-scored FR| in CW and CCW rotations were 1.16 ± 0.05 including 1,152/1,490 neurons and 0.85 ± 0.03 including 1,112/1,490 neurons, respectively; *P* = 4.5e^−3^). Furthermore, more than half of these neurons (56%; 834/1,490 neurons) showed a significant direction preference, responding more strongly to head movements in one direction (Fig. 1*B*, *Materials and Methods*), as previously reported (7). Among this population, neurons preferring CW rotations were slightly yet significantly overrepresented (55%; 455/834 neurons prefer CW rotations; *P* = 3.5e^−3^). To determine whether the preference for CW rotations in left V1 represents hemispheric specialization, we recorded from V1 neurons in the right hemisphere. Neurons in right V1 showed a bias toward CCW head rotation, thus opposite to left V1. Specifically, among right V1 neurons showing significant direction preference, the proportion of neurons preferring CW rotations was significantly lower than in left V1 (45%; 48/106 neurons; *P* = 1.4e^−3^), while the proportion of neurons preferring CCW rotations was similar to that for CW rotations in left V1 (55%; 58/106 neurons; *P* = 0.4). These results reveal that in V1, neurons respond to head movements with a slight overrepresentation for contraversive rotations.

V1 neurons’ response to head movements exhibited diverse amplitudes, kinetics, and signs (Fig. 1*A*), and the first five PCs captured 89% of the variance (Fig. 1*C*; variance per PC ≥ 0.05; *Materials and Methods*). To explore the population dynamics, we visualized activity along the first three PCs (Fig. 1*C*). Before head movement onset, activity was confined to a small neural space. As speed increased, distinct trajectories emerged for CW and CCW movements, consistent with the direction preference of V1 neurons described above. Interestingly, the population dynamics observed with decreasing speed did not overlap with that of increasing speed, and this differential representation persisted for several seconds after the head movement ceased (3.7 s; *P* < 0.05; Fig. 1 *C* and *D*). These results suggest that V1 encodes information about head movements beyond movement direction and instantaneous speed, potentially including changes in speed over time and a trace of previous movements.

To quantify the dependency of each PC on head movement variables, we fitted their dynamics using a model that depends linearly on speed (defined as the absolute value of velocity), angular velocity (CW and CCW rotations as positive and negative velocities), and their derivatives. To capture the long decay time scales of PCs, we convolved each variable with an exponential kernel. This model captured the dynamics of all three PCs (Fig. 1*D*; explained variance ≥ 0.94). While these kinematic variables are correlated in a single movement trajectory, which limits the interpretability of regression coefficients, we also fitted reduced models containing only one variable at a time and compared their predictive performance to the full model (Fig. 1*D*, colored bars). This approach allowed us to assess the contribution of each kinematic variable (see *Materials and Methods* for details). Fitting each variable individually revealed that speed and its derivative explained 86% and 60% of PC1 variance, angular velocity and acceleration explained 75% and 80% of PC2 variance, and speed and its derivative explained 46% and 61% of PC3 variance (see *Materials and Methods* for details and kernel time constants). Thus, V1 population dynamics reflect head angular velocity, speed, and their derivatives.

To provide an independent assessment of kinematic variable representation and to test whether head movement variables could be extracted on a trial-by-trial basis, we developed a decoding analysis. Logistic regression models predicted movement direction (CW or CCW) using neural activity in 100 ms bins (Fig. 1*E*; *Materials and Methods*). With all recorded neurons (n = 2,355), decoding error was at chance before movement onset but dropped below 0.05% within 300 ms of initiation, remaining above chance for 3.7 s after offset. Accuracy exceeded 99.5% with fewer than 600 neurons (Fig. 1*E*), with deeper-layer neurons (below 500 µm) contributing more than superficial ones (above 500 µm). Notably, single high-weight neurons could predict direction with over 99.5% accuracy (*SI Appendix*, Fig. S2*A*). The speed of head movements was accurately decoded from V1 activity (error = 6.8 ± 0.8 deg/s with 2,355 neurons in 100 ms bins), with deeper-layer neurons providing more information (Fig. 1*F*). Unlike direction decoding, where high-weight neurons maintained their responses for seconds postmovement, the neurons decoding speed closely tracked instantaneous head speed, achieving similar accuracy with just ~30 neurons (11.9 ± 2.1 deg/s; *SI Appendix*, Fig. S2*A*). Derivatives of speed, velocity, and acceleration were decoded with similar performance (Fig. 1*F* and *SI Appendix*, Fig. S2 *B* and *C*). Importantly, decoders trained on one head movement profile generalized well to movements with distinct kinematic characteristics (*SI Appendix*, Fig. S2 *D*–*F*), demonstrating that neural representations of head movement variables are not tightly bound to the stereotypy of any single trajectory and reflect fundamental kinematic properties rather than trajectory-specific patterns.

Taken together, these results show that mouse V1, and especially its deeper layers, encodes a rich representation of head movement that can be accessed to simultaneously decode both present and past movements with high precision.

### Pulvinar Origin of Head Movement Signals to V1.

**Fig. 2.**
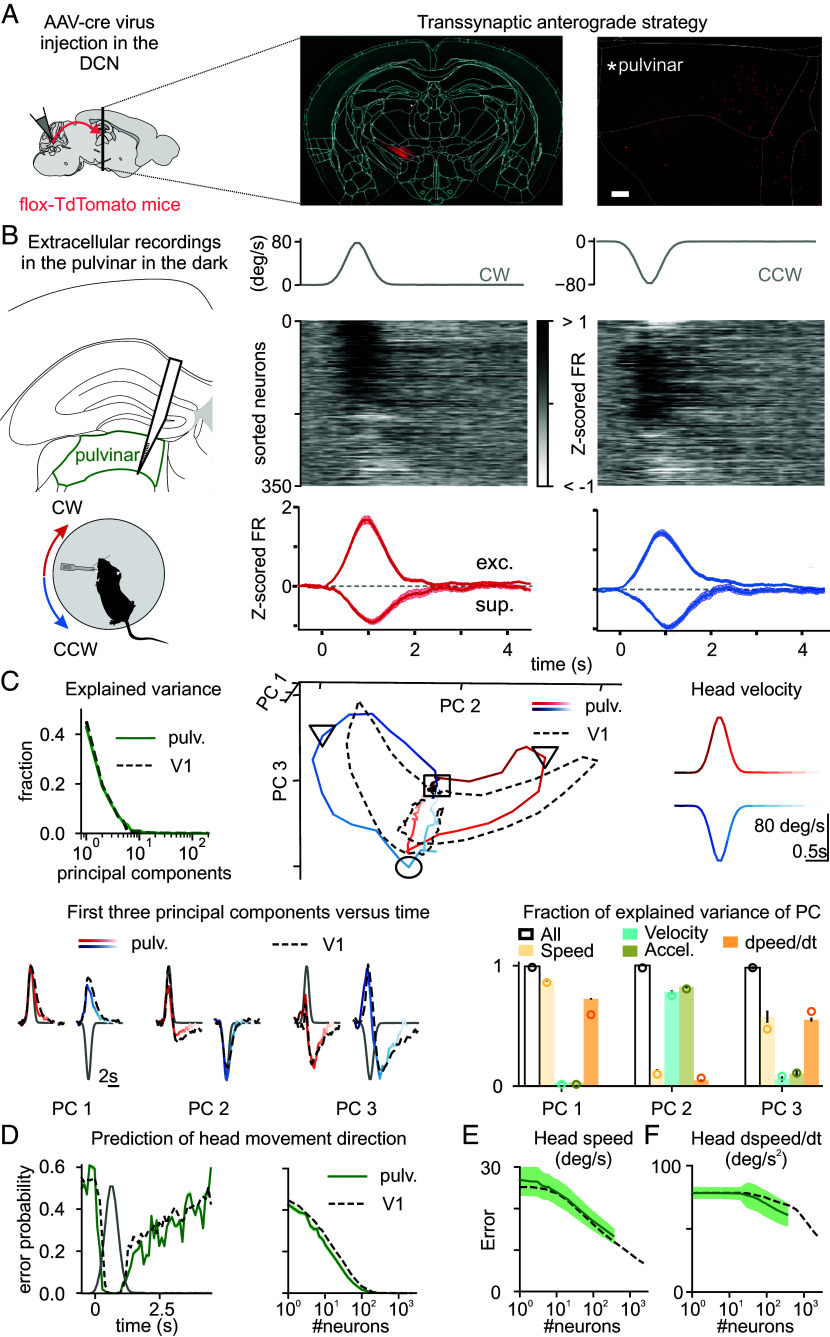
The pulvinar receives input from the DCN and encodes head movements like V1. (*A*) Experimental strategy. (*Left*) Injection of Cre-dependent transsynaptic anterograde virus (AAV1 hSyn-cre) in the DCN in a flex-tdTomato reporter line; this approach labels the projections of the DCN and the postsynaptic neurons in red (*Materials and Methods*). (*Middle*) Photomicrograph of coronal sections at −1.98 mm from Bregma illustrating transynaptically labeled neurons. (*Right*) Zoom on the left pulvinar thalamus. (Scale bar, 100 μm) (*B*) Experimental configuration. (*Left*) Extracellular linear probe in the left pulvinar of a head-fixed, awake mouse records the response to CW (red) and CCW (blue) rotations of the table in the dark. (*Right*) UMAP sorting of the averaged Z-score of the FR (Z-scored FR) responses across neurons (neurons were ordered using UMAP with cross-validation), and Z-scored FR of pulvinar neurons that significantly respond to CW (red) and CCW (blue) head movements. The gray traces on top are the velocity profile. (*C*) Comparison of principal component (PC) analysis in pulvinar and V1. (*Top Left*) Fraction of explained variance in the pulvinar (green) and in V1 (black, as described in Fig. 1*D*). (*Top Right*) Three-dimensional representation of the first three PCs for pulvinar activity (time is color coded: CW (red) and CCW (blue) rotations; square, triangle, and circle symbols indicate beginning, peak, and end of the rotation, respectively) and V1 activity (dashed lines). (*Bottom*) Variance explained in each PC by models including speed, velocity, and their time derivative either all together (empty bar) or separately (color). Circles are the fraction of explained variance in V1 (from Fig. 1*D*). (*D*) Decoding error probability of head movement direction in a trial as a function of time (*Left*) and number of neurons (*Right*, computed at peak velocity) in pulvinar (green) and V1 (black). (*E*) Decoding error of head angular speed as a function of number of neurons in pulvinar (green) and V1 (black). (*F*) As in *E* but for head angular derivative of speed in time.

What are the sources of the head movement signals that reach V1? To identify potential upstream candidate areas, we injected an anterograde transsynaptic tracer in the DCN, one of the main sources of vestibular signals to the brain. Injection of AAV2/1-hSyn-Cre in the DCN of a tdTomato reporter mouse labeled neurons throughout the thalamus, in agreement with previous work (12–14). Among visual areas, only the pulvinar nucleus of the thalamus receives direct DCN projections (Fig. 2*A* and *SI Appendix*, Fig. S3 *A* and *B*). In contrast, DCN injections labeled very few neurons in the dorsal lateral geniculate nucleus (dLGN), the other main thalamic relay to the VC, nor did they label neurons in V1 (pulvinar: 896.5 ± 97.8 per mm^3^; dLGN: 27.8 ± 5.7 per mm^3^, *P* = 6.6e^−17^; no labeled neurons in V1; N = 8 mice; *SI Appendix*, Fig. S3*B*), consistent with the lack of direct projection from the DCN to the cortex (12–14). Interestingly, we found that only the rostral part of the pulvinar contains neurons receiving DCN projections (*SI Appendix*, Fig. S3 *A* and *B*). Specifically, we report a much higher percentage of neurons receiving DCN projections in the medial compared to the lateral part of the rostral pulvinar (87.68% ± 1.35% versus 12.32% ± 1.35%, respectively; P = 1.55e^−4^). We confirmed the specificity of the DCN projections to the pulvinar using a retrograde tracer. Injection of retrograde AAV-Cre in the rostro-medial pulvinar of tdTomato reporter mice labeled more neurons in the DCN than in the other main source of vestibular signals—the vestibular nuclei (DCN: 682.4 ± 82.7 per mm^3^; VN: 94.5 ± 27.0 per mm^3^; *P* = 2.7e^−11^; N = 3 mice; *SI Appendix*, Fig. S3 *C* and *D*). Thus, rostro-medial pulvinar neurons receive direct projections from the DCN, making this thalamic nucleus a potential node for vestibular signals on their way to V1.

If the pulvinar is a source of head movement signals to the VC, it must respond to head movements. Thus, we recorded neuronal activity from the left pulvinar of head-fixed, awake mice in response to vestibular stimulation delivered in the dark by rotating the animal along the horizontal plane (Fig. 2*B*), as we did for V1 recordings (see above). Similar to V1, the FR of most pulvinar neurons (73%; 258/355 neurons; N = 11 mice) was modulated by CW (58%; 207/355 neurons) and/or CCW (51%; 182/355 neurons) rotations of the table and a large fraction of these neurons (45%; 160/355 neurons) exhibited a significant direction preference (Fig. 2*B*). Furthermore, the response of left pulvinar was also biased toward CW head movements (average Z-scored FR for CW 1.36 ± 0.10 and CCW 1.04 ± 0.08; *P* = 3.7e^−3^). In fact, the pulvinar showed a larger fraction of neurons preferring CW rotations than V1 (62% including 100/160 neurons in the pulvinar versus 55% including 455/834 neurons in V1; *P* = 3.7e^−2^), corresponding to an absolute difference of eight percentage points and a small-to-moderate effect size (Cohen’s h = 0.16). Finally, principal component analysis of pulvinar activity during head movement revealed a remarkable similarity with that observed in V1 (Fig. 2*C*). Specifically, the activity along the first three PCs (Fig. 2 *C*, *Top Right* and *Bottom*) closely matched those observed in V1. Moreover, the first three PCs were explained by head movement-related variables in an analogous way to V1 (Fig. 2*C*). The similarity between head movement representation properties in the pulvinar and V1 was equally striking when quantified with a decoding analysis. Decoding performances of trial head movement direction as a function of time (Fig. 2*D*), as well as of speed (Fig. 2*E*), velocity (*SI Appendix*, Fig. S3*F*), and their time derivatives (derivative of speed: Fig. 2*F*; acceleration: *SI Appendix,* Fig. S3*G*), closely resemble what we observed in V1.

Taken together, these results demonstrate that head movements modulate the activity of a large fraction of pulvinar neurons to generate a rich representation that matches the one observed in V1, albeit with a stronger bias toward contraversive movements. Thus, the pulvinar is a potential source of vestibular signals upstream of V1.

**Fig. 3.**
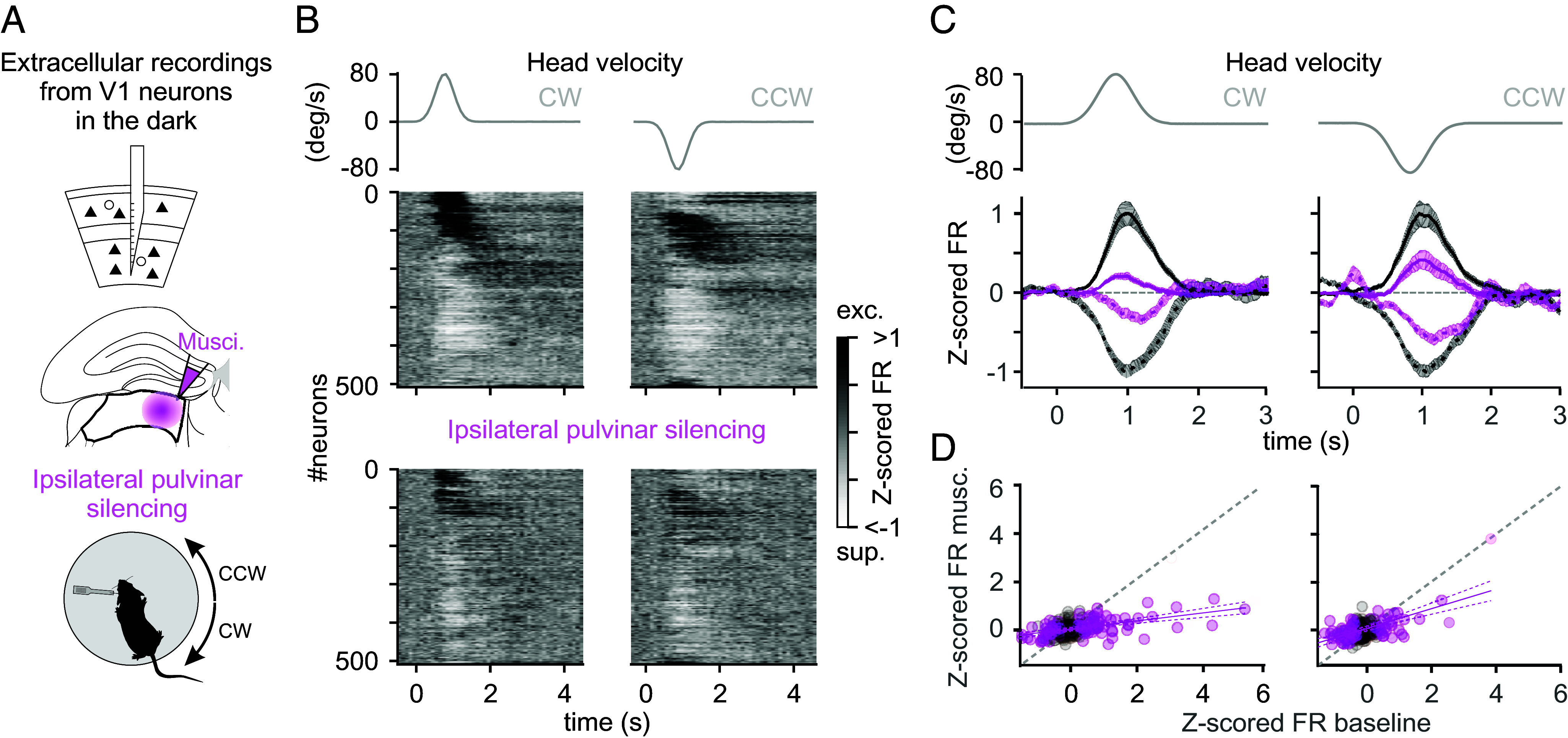
V1’s response to head movements depends on the ipsilateral pulvinar. (*A*) Experimental configuration. (*Top*) Linear probe spanned all cortical layers, before and after ipsilateral pulvinar silencing by injecting muscimol-BODIPY. (*Bottom*) Extracellular linear probe in the left V1 of a head-fixed, awake mouse records the response to CW and CCW rotations of the table in the dark. (*B*) UMAP sorting of the averaged Z-scored FR of V1 neurons. (*Left*) In response to CW head movements. (*Right*) In response to CCW head movements. (*Middle*) In control condition before pulvinar silencing. (*Bottom*) After pulvinar silencing. Neurons were ordered so nearby ones had similar Z-scored FR over time (UMAP with cross-validation; see *Materials and Methods*). The gray traces on top are the velocity profile. (*C*) Peak normalized Z-scored FR of V1 neurons. (*Left*) In response to CW head movements. (*Right*) In response to CCW head movements. In control condition (dark traces) and after ipsilateral pulvinar silencing (magenta traces). Note that for *C* and *D*, only neurons significantly modulated during head movements under control condition contribute to each average depending on whether they were excited (solid lines) or suppressed (dashed lines) by the rotation. All traces are normalized by the peak of the Z-scored FR under control condition. The gray traces on top are the velocity profile. (*D*) The peak Z-scored FR. (*Left*) In response to CW head movements. (*Right*) In response to CCW head movement. Control condition (*x*-axis) versus pulvinar silencing (*y*-axis). Magenta circles represent neurons that significantly respond to head movements under control conditions. Gray circles represent neurons that do not significantly respond to head movement under control conditions.

To determine whether the pulvinar contributes to the representation of head movements in V1, we pharmacologically silenced the left pulvinar via the stereotactic injection of the fluorescently labeled GABAergic agonist muscimol-BODIPY while simultaneously recording V1 activity in response to vestibular stimulation. Post hoc analysis confirmed that muscimol-BODIPY diffusion was mainly confined within the pulvinar (*SI Appendix*, Fig. S4; *Materials and Methods*). Muscimol-BODIPY injection resulted in a slight decrease in the basal activity of V1 neurons [control: FR = 3.77 ± 0.29 Hz (average ± SEM); pulvinar silencing: FR = 3.44 ± 0.26 Hz; *P* = 1.0e^−3^; n = 358 neurons, N = 4 mice], yet strongly reduced their response to head movements (CW: 72 ± 5% decrease of Z-scored FR; *P* = 1.5e^−8^; CCW: 54 ± 6% decrease of Z-scored FR; *P* = 1.7e^−5^; Fig. 3 *B* and *C*). This effect was more pronounced for CW rotations (CW versus CCW: *P* = 6.0e^−3^, Fig. 3*C*), consistent with the biased representation of CW head movements in the pulvinar (see above). Consistent with this result, decoding analyses revealed that the accuracy of the decoding of head movement direction from V1 activity was significantly reduced following pulvinar silencing, with a stronger drop for CW compared to CCW rotations (*SI Appendix*, Fig. S5*A*).

Taken together, these results indicate that the pulvinar is a main source of vestibular signal to V1 with a bias toward contraversive head movements.

### Contralateral VC Contribution of Head Movement Signals to V1.

The fact that silencing the left pulvinar reduces responses in left V1 to CW more than to CCW rotations suggests that left V1 receives CCW head movement signals from an additional source. Because responses in right V1 are biased toward CCW rotations (see above), and V1 hemispheres are connected via transcallosal projections (15, 16), we tested the potential contribution for right V1 to CCW head movement signals in left V1.

**Fig. 4.**
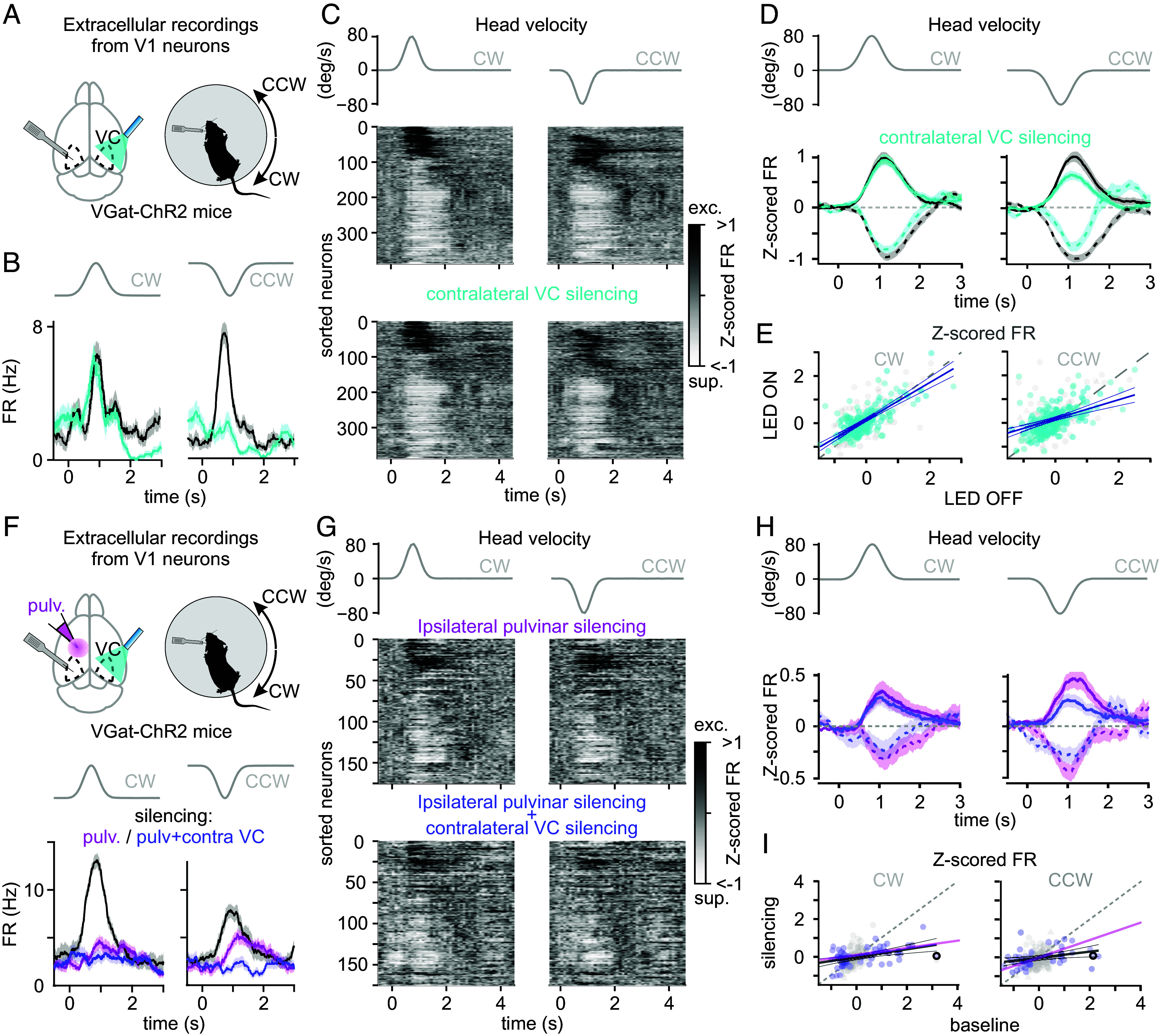
Ipsilateral pulvinar and contralateral VC are sources of complementary vestibular signals to V1. (*A*) Experimental configuration. (*Left*) Extracellular linear probe positioned in the left primary VC (V1) while optogenetically silencing the contralateral VC using LED illumination through a thinned skull on a VGat-ChR2-EFP transgenic mouse. (*Right*) Extracellular linear probe in the left V1 of a head-fixed, awake mouse records the response to CW and CCW rotations of the table while optogenetically silencing the contralateral VC. (*B*) Effect of silencing contralateral VC on an example neuron recorded in left V1. Z-scored FR. (*Left*) In responses to CW head movements. (*Right*) In responses to CCW head movements. Control condition (dark traces) and during contralateral VC silencing (cyan traces). The gray traces on top are the velocity profile. (*C*) UMAP sorting of the averaged Z-scored FR of V1 neurons. (*Left*) In responses to CW head movements. (*Right*) In response to CCW head movements. (*Middle*) In control condition. (*Bottom*) During contralateral VC silencing. The gray traces on top are the velocity profile. (*D*) Peak normalized Z-scored FR of V1 neurons. (*Left*) In responses to CW head movements. (*Right*) In responses to CCW head movements. Control condition (dark traces) and during contralateral VC silencing (cyan traces). (*E*) Relation between Z-scored FR in control versus contralateral VC silencing conditions. Same neurons as in *D*. (*F*) Experimental configuration. (*Left*) Extracellular linear probe positioned in the left primary VC (V1) before and after pharmacological silencing of the ipsilateral pulvinar (muscimol-BODIPY), while the contralateral optogenetically VC (same as *A*) is silenced on alternate trials. (*Right*) Extracellular linear probe in the left V1 of a head-fixed, awake mouse records the response to CW and CCW rotations of the table while silencing the ipsilateral pulvinar and the contralateral VC. (*Bottom*) Effect of silencing contralateral VC and ipsilateral pulvinar on an example neuron. Z-scored FR of a V1 neuron. (*Left*) In response to CW. (*Right*) In response to CCW head movements. In control condition (dark traces), during ipsilateral pulvinar silencing (magenta traces), and during simultaneous ipsilateral pulvinar and contralateral VC silencing (purple traces). (*G*) UMAP sorting of the averaged Z-scored FR of V1 neurons. (*Left*) In responses to CW head movements. (*Right*) In response to CCW head movements. (*Top*) After ipsilateral pulvinar silencing only. (*Bottom*) After both ipsilateral pulvinar and contralateral VC silencing. The gray traces on top are the velocity profile. (*H*) Peak normalized Z-scored FR of V1 neurons. All traces are normalized by the peak Z-scored FR observed under control condition. (*Left*) In response to CW head movements. (*Right*) In response to CCW head movements. Following ipsilateral pulvinar silencing (magenta traces) and during contralateral VC and pulvinar silencing (purple trace). The gray traces on top are the velocity profile. Note that the control condition is not represented. (*I*) Relation between Z-scored FR in control versus simultaneous contralateral VC silencing and ipsilateral pulvinar silencing conditions. Bold circles report the example neuron in *F*. Note that for *D* and *H*, only neurons significantly modulated during head movements in the control condition contribute to each average depending on whether they were excited (solid line) or suppressed (dashed line) by the rotation. All traces are normalized by the peak Z-scored FR observed in control condition. The gray traces on top are the velocity profile.

For this, we optogenetically silenced the right VC while recording from left V1 (Fig. 4*A*). Silencing was achieved by photoactivating inhibitory neurons expressing Channelrhodopsin2 and the enhanced yellow fluorescent protein (ChR2-EYFP; VGat-ChR2-EYFP mouse line) with a light-emitting diode (LED) placed on top of the right VC, as described previously (17–19). Silencing trials were alternated with control ones without LED illumination. We prevented LED-evoked visual responses (i.e., LED light hitting the retina) to affect our V1 recordings by performing experiments in mice previously blinded by intraocular Tetrodotoxin (TTX) injections in both eyes (*Materials and Methods*). LED illumination led to a slight decrease in the average basal activity of left V1 neurons [control FR = 2.78 ± 0.20 Hz (average ± SEM); contralateral VC silencing FR = 2.70 ± 0.19 Hz; *P* = 1.8e^−10^; n = 408 neurons; N = 7 mice; 23% (95/408) of the neurons were suppressed and 19% (75/408) excited]. Strikingly, silencing the right VC selectively reduced responses to CCW head rotations in left V1 (33.2 ± 5.7% decrease of Z-scored FR; *P* = 8.0e^−10^) leaving responses to CW rotations unaffected (CW: 11.6 ± 5.7% decrease of Z-scored FR; *P* = 0.83; CW versus CCW: *P* = 1.0e^−4^; Fig. 4 *A*–*E*). Consistent with this asymmetry, decoding analyses showed a significant reduction in accuracy for both directions following contralateral V1 silencing, with a more pronounced drop for CCW compared to CW rotations (*SI Appendix*, Fig. S5*B*). Thus, the right VC is a source of CCW head movement signals to left V1.

If the ipsilateral pulvinar and the contralateral cortex independently contribute to head movement responses in V1, CCW responses remaining in left V1 after pulvinar silencing should be further reduced by silencing the right VC. We tested this possibility by combining pharmacological silencing of the pulvinar with the optogenetic silencing of the VC. Consistent with this hypothesis, optogenetic silencing of the right VC performed following the pharmacological silencing of the left pulvinar selectively reduced the remaining responses to CCW rotations in left V1 (CW: 14.3 ± 14.5% decrease of Z-scored FR; *P* = 0.61; CCW: 25.0 ± 11.2% decrease of Z-scored FR; *P* = 0.03; n = 175 neurons; N = 5 mice; Fig. 4 *F*–*H*). Moreover, decoding performance was further degraded when both structures were silenced (*SI Appendix*, Fig. S5*C*).

Taken together, these results indicate that the ipsilateral pulvinar is the main contributor to V1 responses to head movements, with a bias toward contraversive rotations while the contralateral VC contributes to responses to ipsiversive rotations. Thus, the ipsilateral pulvinar and, to a lesser extent, the contralateral VC are two independent sources of vestibular signals to V1, each preferentially contributing to responses in opposite directions along the horizontal plane.

## Discussion

Primary sensory areas in the cerebral cortex process modality-specific sensory information originating from peripheral receptors. Recent studies, however, have challenged this strict, modality-specific view (7, 10, 20–24). A striking example is that V1 responds robustly to head rotations via vestibular organ activation (7, 8), even in complete darkness. Thus, V1 is a primary sensory cortical area that not only receives ascending input from the eyes and responds to visual stimuli but also responds to vestibular stimuli. This observation has opened several fundamental questions, relative to the anatomical pathways taken by these vestibular signals to reach V1, the nature of the representation of head movement variables in V1, and the extent to which the representation of these variables is inherited from upstream structures. Here, we demonstrate that V1 accurately encodes head movement variables (direction, speed, velocity, and their time derivatives), especially in deeper layers, and that it receives head movement signals through two main pathways: the ipsilateral pulvinar nucleus of the thalamus and the contralateral VC. The ipsilateral pulvinar provides the predominant head movement signal, exhibiting a bias toward contraversive rotations (e.g., CW rotations relative to left V1). In contrast, the contralateral VC contributes head movement signals during ipsiversive rotations. Importantly, we found that head movement variables in V1 are already represented in the pulvinar, suggesting that V1 inherits these variables rather than computing them ex novo. These results demonstrate that the integration of intra- and interhemispheric signals endows V1 with a rich and accurate representation of head movements.

Sensory stimuli often trigger behavioral responses, which, in turn, can lead to a cortex-wide modulation of neuronal activity (21, 25). Consequently, some activity in primary cortical areas in response to stimuli of distinct modalities may be erroneously interpreted as multimodal responses. For example, a substantial portion of V1 neurons’ response to auditory stimuli can be explained by facial movements triggered by the sound, and are thus not true auditory V1 responses (23, 24). In our experiments, head movements also triggered a behavioral response, namely compensatory eye movements through the activation of the VOR (26). Given that the kinetics and amplitudes of both head and eye movements are tightly linked during VOR, V1 responses to head movement could, in theory, be explained by eye rather than head movements. This possibility would be consistent with the fact that the VOR, like the response of V1 to head movements (7, 8), depends on the vestibular organ (27). To address this possibility, we used a well-established protocol to eliminate eye movements during head movement, called VOR cancellation (28). Even with this protocol, head movements generated strong responses in V1. While these data demonstrate that head movements in the absence of eye movements trigger robust V1 responses, they do not exclude a possible contribution of compensatory eye movements to V1 activity during VOR.

The thalamus receives the majority of its vestibular input from the contralateral vestibular and cerebellar nuclei (12–14), and these nuclei are primarily biased toward ipsiversive movements. Consistent with this organization, we observed a contraversive movement bias in the pulvinar (i.e., CW for the left pulvinar). However, this contraversive bias of the pulvinar almost disappears in V1. We found that this rebalancing of motion specificity is due to input from the contralateral VC. While the cortico-callosal projections in the binocular V1 have been well characterized (29), their role in monocular V1 is still unclear (15, 16). Recent work has shown that excitatory L6 corticocortical neurons give rise to a major pathway linking the two hemispheres (15, 16). Here, we show that this interhemispheric communication to monocular V1 provides a head movement signal with a direction preference bias opposite to that obtained from the ipsilateral pulvinar. These results suggest that interhemispheric projections, possibly originating from L6, are biased toward contraversive rotations. Whether this rebalancing is critical for a complete (ipsi- and contralateral) picture of motion or whether the segregation and recombination of motion signals serves a specific computational purpose remains to be seen.

Several pathways connect the vestibular system to the primate’s thalamus (30), and accordingly, the pulvinar of these animals responds to various vestibular stimuli (31). Furthermore, the pulvinar receives projections from motor areas, including the motor cortex and DCN (12, 13, 32), and projects broadly across the VC, conveying not only visual information but also other nonvisual inputs (10, 33). Interestingly, inactivating the pulvinar abolishes the saccadic modulation in V1 neurons (10), highlighting the pulvinar’s role in integrating both visual and nonvisual information. Our results demonstrate that, in mice, the rostral-pulvinar serves as the primary source of head movement signals to V1. These findings align with the established anatomical organization of pulvinar subregions in primates (34). The rostro-medial pulvinar represents a functionally distinct compartment that, unlike the posterior pulvinar (which receives predominant superior colliculus input), integrates projections from V1, frontal cortical areas (35, 36), and—as demonstrated here—the DCN. This connectivity pattern supports our hypothesis of visual-vestibular integration within the rostro-medial pulvinar, consistent with recent anatomical mapping studies (12, 13, 37). The convergence of visual information in V1 with head and eye movement signals conveyed through the pulvinar could contribute to mechanisms for maintaining stable perception during active behavior. Indeed, the pulvinar has recently emerged as critical for distinguishing self-generated from external visual motion (10), and its role in predictive coding (38) may reflect a broader strategy where the brain uses movement-related signals to anticipate sensory consequences. Future work will reveal how the pulvinar’s diverse functions arise from its unique position between motor and sensory systems, and how this integration shapes our perception of a stable visual world during self-motion.

Although the pulvinar projects directly to V1, the functional pathway connecting these two structures may involve multiple routes. The pulvinar also sends projections to other cortical regions, including higher visual areas and retrosplenial cortex (RSC)—both of which are modulated by head motion and project to V1. This anatomical organization suggests that vestibular signals from the pulvinar could reach V1 through both direct and indirect pathways. Indeed, recent work indicates a contribution of RSC to vestibular signals in V1 (8). We cannot exclude a contribution of other thalamic nuclei such as the latero-dorsal (LD) thalamus, to the vestibular signals recorded in V1. However, the fraction of the vestibular signal abolished upon injection of muscimol in the rostral pulvinar cannot be attributed to the concomitant silencing of adjacent nuclei (e.g., LD), because of the precise targeting and minimal spread of muscimol in these experiments (*Materials and Methods*). Of the thalamic nuclei that project to the VC, DCN neurons target primarily the rostro-medial pulvinar. This selective targeting of DCN projections to a subregion of the pulvinar is consistent with the idea that, like the primate pulvinar, also the rodent homolog is specialized into functionally and anatomically distinct areas. The secondary motor cortex (M2) has also been shown to contribute to head motion signals in V1 (39) in the context of self-generated head movement in freely moving rodents. Given that the rostro-medial pulvinar exhibits robust reciprocal connectivity with M2 areas [Anterior cingulate cortex and Orbitofrontal cortex; (32, 35, 40)], the pulvinar may serve as a critical integration center that receives signals related to both self-generated and externally generated head movements. Thus, in addition to the rostro-medial pulvinar V1, may receive head motion signals from other pathways, and these pathways could differ depending on whether head movements are self- or externally generated.

In summary, the intra- and interhemispheric vestibular signals to V1 described here may impact cortical visual processing by providing a head movement context for incoming visual inputs. Understanding the origin and processing of these signals could offer critical insights into how the early visual system integrates visual input with contextual information related to motion, thereby enhancing our understanding of sensory processing during action.

## Materials and Methods

### Experimental Model and Subject Details.

#### Mice.

All experimental procedures were conducted in accordance with the regulations of the Institutional Animal Care and Use Committee (AN179056) of the University of California, San Francisco. All mice were housed on a reversed cycle (light/dark cycle 12/12 h) with free access to food. Data were collected from male or female C57BL/6J mice or from heterozygous mice kept on a C57BL/6J background with the following genotype: VGat–ChR2–EYFP (Jackson Labs #014548). V1 recordings in darkness included 1,502 units from 30 C57BL/6J mice, obtained from our previous study under identical experimental conditions (7). At the start of the experiments, all mice were between 2 and 7 mo old.

Method details can be found in *SI Appendix*.

## Supplementary Material

Appendix 01 (PDF)

## Data Availability

Analysis code and data are available on Zenodo (https://doi.org/10.5281/zenodo.17154402) (41) and GitHub (https://github.com/sanzeni/vestibular-v1-sources) (42). All other data are included in the article and/or *SI Appendix*.
