# Supplementary material for: Inter- and intrahemispheric sources of vestibular signals to V1
Source: Proc Natl Acad Sci U S A. 2025 Oct 10;122(41):e2503181122. doi: 10.1073/pnas.2503181122 (PMC12541342; doi:10.1073/pnas.2503181122)
Supplement: Supplementary file 1 — Appendix 01 (PDF) [file pnas.2503181122.sapp.pdf]

Inter- and Intrahemispheric Sources of Vestibular Signals to V1

Guy Bouvier[1,2,3,*,#], Alessandro Sanzeni[4,5,6,*], Elizabeth Hamada[7], Nicolas Brunel[4,6], & Massimo Scanziani[1,2,#]

Affiliations
[1] Department of Physiology, University of California, San Francisco, San Francisco, CA, USA
[2] Howard Hughes Medical Institute, University of California, San Francisco, San Francisco, CA, USA
[3] Université Paris-Saclay, CNRS, Institut des Neurosciences Paris-Saclay, 91400 Saclay, France
[4] Department of Computing Sciences, Bocconi University, 20100 Milan, Italy
[5] Center for Theoretical Neuroscience and Mortimer B Zuckerman Mind Brain Behavior Institute, Columbia University, New York, NY 10027, USA
[6] Department of Neurobiology, Duke University, Durham, NC 27710, USA
[7] Department of Neurology, University of California, San Francisco, San Francisco, CA, USA
* The authors contributed equally to this work.

[#] **Corresponding authors**: massimo@ucsf.edu and guy.bouvier@cnrs.fr

SUPPORTING INFORMATION

MATERIALS AND METHODS

EXPERIMENTAL MODEL AND SUBJECT DETAILS

Mice

All experimental procedures were conducted in accordance with the regulations of the Institutional Animal Care and Use Committee (IACUC, AN179056) of the University of California, San Francisco. All mice were housed on a reversed cycle (light/dark cycle 12/12 h) with free access to food. Data were collected from male or female C57BL/6J mice or from heterozygous mice kept on a C57BL/6J background with the following genotype: VGat–ChR2–EYFP (Jackson Labs #014548). V1 recordings in darkness included 1,502 units from 30 C57BL/6J mice, obtained from our previous study under identical experimental conditions (1). At the start of the experiments, all mice were between 2 and 7 months old.

METHODS DETAILS

Viruses

The following adeno-associated viruses (AAV) were used: AAV1-hSyn-Cre-WPRE-hGH (final titer: 1.8×10^{13} genome copies/ml, Univ. of Pennsylvania Viral Vector Core) and AAV1-retro-hSyn-Cre-eBFP (final titer: 5×10^{12} genome copies/ml, Univ. of Pennsylvania Viral Vector Core).

Surgical procedures

Viral Injections*:* Mice were anesthetized with 2% isoflurane and placed in a stereotactic apparatus (Kopf). Core body temperature was monitored with a rectal probe and maintained constant at 37°C with a heating pad (FHC). A thin layer of lubricant ointment (Rugby Laboratories) was applied to the eye, the head was shaved and disinfected with povidone iodine, and 2% lidocaine solution was administered subcutaneously at the incision site. A craniotomy (approx. 300 μm in diameter) was performed with a micro-burr (Gesswein) mounted on a dental drill (Foredom). Viral suspensions were loaded in beveled glass capillaries (tip diameter: 15-30 μm) and injected with a micropump (UMP-3, WPI) at a rate of 30-40 nl/min into the parenchyma. The coordinates of the injection sites and the volumes of the injected viral suspension are detailed below. The pipette was removed from the brain 15 min after the completion of the injection, the head plate was attached just after the virus injection, and 0.1 mg/kg buprenorphine was administered subcutaneously as a postoperative analgesic. For anterograde transsynaptic strategy (2), the virus was injected in the right DCN (AP: -6.1 mm; ML: 2.0 mm; depth: 2.25 mm; volume = 100-250 nl). For retrograde strategy in the pulvinar, the virus was injected in the left rostro-medial pulvinar (AP: -1.9 mm; ML: 1 mm; depth: 2.4 mm; volume = 30-50 nl).

Head Plate Implantation for Head-fixed Recordings*:* Mice were implanted with a T-shaped head-bar at least 2.5 weeks before the day of the recording. Mice were anesthetized with 2% isoflurane, the scalp was removed, the skull was disinfected with alcohol and povidone iodine, and scored with bone scraper. The edge of the skin was glued to the skull and the metal head-bar was sterilized and mounted using dental cement (Ortho-Jet powder; Lang Dental) mixed with black paint (iron oxide), or Relyx Unicem2 automix (3M ESPE). The head-bar was stereotactically mounted with the help of an inclinometer (Digi-Key electronics 551-1002-1-ND). The inclinometer allowed us to adjust the angle of the head bar in relation to the sagittal and medio-lateral axes of the head. Following the bar implantation, black dental cement was used to build a recording well surrounding the recording site. The surface of the skull above the left visual cortex was not covered with dental cement but was coated with a thin layer of transparent cyanoacrylate glue. Mice were injected subcutaneously with 0.1 mg/kg buprenorphine and checked daily after the head-bar surgery. For at least 4 days before recording, mice were habituated to head fixation within the recording setup.

Craniotomy for Electrophysiological Recordings*:* On the day before recording, mice were anesthetized with 2% isoflurane and the skull above the recording sites was drilled off. The dura was not removed, and the exposed brain was kept moist with artificial cerebrospinal fluid (ACSF; 140 mM NaCl, 5 mM KCl, 10 mM D-glucose, 10 mM HEPES, 2 mM $CaCl_2$, 2 mM $MgSO_4$, pH 7.4). V1 recordings were performed at approximately 2.6 mm lateral to the sagittal suture and 0.6 mm anterior to the lambdoid suture.

Electrophysiology

Extracellular recordings were performed using the following silicon probes Neuronexus: A1x32-5mm-25-177-A32; A1x32-Edge5mm-20-177-A32; A2x32-5mm-25-177-A64, 1x64-Poly2-6mm-23 s-160 or Cambridge Neurotech: ASSY-77 H2 (Acute 64 channel H2 probe, 2 shanks @250 μm, 8 mm length), ASSY-77 H5 (Acute 64 channel H5 probe, 1 shank, 9 mm length). The recording electrodes were controlled with Luigs & Neumann micromanipulators

and stained with DiI or DiO lipophilic dyes (Thermo Fisher) for post hoc identification of the electrode track. V1 coordinates were confirmed *post hoc* and all the pulvinar recordings were performed in the rostro-medial pulvinar (antero-posterior coordinates: -1.75 to -2.1 mm from Bregma; medio-lateral coordinates: 0.7 to 1.3 mm from midline). The choice of these coordinates was motivated by our tracing experiments showing that the deep cerebellar nuclei project to the rostral part of the pulvinar. We recorded the signals at 30 kHz using an INTAN system (RHD2000 USB Interface Board, INTAN Technologies).

Head-fixed Rotations

To control the velocity and amplitude of head movements, we fixed the head of awake mice in the center of a servo-controlled platform enabling the rotation of the animal along the horizontal plane (50 degrees rotation; 80 deg/s peak velocity, see Fig. 1A; unless stated otherwise). Mice were head-fixed, their bodies restrained in a tube, and we pseudo-randomly alternated clockwise (CW) with counterclockwise (CCW) rotations. The platform was attached to a gearbox 15:1 (VTR010-015-RM-71 VTR, Thomson) that increased the torque of a servo motor (AKM53L-ANC2C-00 KEC0432 AC Servomotor 1.83 kW, Kollmorgen). The motor was tuned using a servo drive (AKDB013206-NBAN-0000 servo drive, Kollmorgen) and controlled in velocity mode using analog waveforms computed in Labview.

Monitoring eye movements by video-oculography

The movement of the right eye was monitored through a high-speed infrared (IR) camera (Imperx Bobcat, B0620). The camera captured the reflection of the eye on an IR mirror (transparent to visible light, Edmund Optics #64–471) under the control of custom Labview software and a frame grabber (National Instrument PCIe-1427). The pupil was identified online or post hoc by thresholding pixel values and its profile was fitted with an ellipse to determine the center. The eye position was measured by computing the distance between the pupil center and the corneal reflection of a reference IR LED (940nm) placed along the optical axis of the camera. To calibrate the measurement of the eye position, the camera and the reference IR LED were moved along a circumference centered on the image of the eye by ± 10 degrees (3). Note that simultaneous eye tracking was performed only in vestibulo-ocular reflex experiments.

Vestibulo-ocular reflex paradigms

To assess vestibulo-ocular reflex (VOR) compensation and cancellation, well-habituated mice were head-fixed on a rotating platform surrounded by a visual virtual stimulus drum[36]. We presented visual stimuli (0.1 cpd) moving synchronously with the turntable (20 deg peak velocity, 1.8 s, 15 deg) during VOR cancellation and static during VOR compensation trials. Eye movements were tracked as described above. During VOR cancellation trials, the platform and visual drum were rotated using a gaussian velocity waveform in the same direction and at matching velocities. This condition required mice to suppress their VOR to maintain a stable gaze on the moving visual stimulus. Eye position data were analyzed offline using custom MATLAB scripts to calculate gain (ratio of eye and head velocity). VOR cancellation performance was quantified as the absence of reflexive eye movement, while expecting a reflexive eye movement in the opposite direction during VOR compensation. Rapid eye movements were excluded from our analysis.

Pharmacology

Intraocular injection of tetrodotoxin (TTX; 40 μM) was performed 2 hours prior to recording, under isoflurane anesthesia. A typical procedure lasted less than five minutes. TTX was injected in both eyes for all the experiments performed on VGat-ChR2-EYFP mice. Immediately prior to the injection, a drop of proparacaine hydrochloride ophthalmic solution was applied to the eye as a local anaesthetic (Bausch + Lomb, 0.5%). TTX solution was injected intravitreally using a beveled glass micropipette (tip diameter ~50 μm) on a micro injector (Nanoject II, Drummond) mounted on a manual manipulator. 1 μl was injected in each eye, at the speed of 46 nl/s. The animals were head-fixed for recording following a 2-hour recovery period in their home cage.

Silencing of the pulvinar was performed by injecting 30-40 nl of 5 mM muscimol-BODIPY at the speed of 80-150 nl/min, using a beveled glass pipette (tip diameter ~ 20-40 μm) on a micro injector UMP3 with a Micro4 controller (World Precision Instruments). The injector was mounted on a micromanipulator (Luigs & Neumann) for stereotactic injection. After the recording, brains were fixed in 4% PFA in PBS overnight at 4°C for histological analysis of BODIPY on the next day. To assess the specificity of muscimol silencing, we quantified the spread of muscimol-BODIPY in the anterior-posterior axis from the injection site in the rostro-medial pulvinar to the adjacent LD thalamus (*SI Appendix*, Fig. S4). We compared fluorescence at the injection site with the fluorescence at the pulvinar-LD border. The fluorescence at the pulvinar-LD border was only 1.18% of that at the injection site. We also quantified the mediolateral spread of muscimol-BODIPY using the same method. We measured fluorescence intensity at the injection site and compared it to fluorescence in the rostro-lateral pulvinar on the same brain slice. The signal in the lateral region reached only 13.27% of the peak fluorescence at the injection site, indicating limited diffusion. Nevertheless, we cannot exclude the possibility that this low concentration may have exerted an effect on the rostro-lateral pulvinar activity.

To verify the absence of visual responses following intraocular TTX injection, we used a full-field luminance change from 0 $cd.m^{-2}$ to 100 $cd.m^{-2}$ lasting 1 s.

Optogenetic silencing of contralateral visual cortex

Cortical silencing was achieved by expressing channelrhodopsin-2 (ChR2) in inhibitory neurons, a technique that has been previously validated (4–6). We utilized the VGat–ChR2–EYFP mouse line for optogenetic silencing of the contralateral visual cortex. For photostimulation of ChR2-expressing cortical inhibitory neurons, we positioned a 470-nm blue fiber-coupled LED (1 mm diameter, Doric Lenses) approximately 5-10 mm above a thinned skull area on the right hemisphere of the visual cortex. To limit illumination to the tissue under the cranial window, we covered adjacent areas with black dental cement. To prevent inadvertent retinal stimulation from blue light, we induced temporary blindness by injecting both eyes with TTX (see above). The LED fiber delivered a total light power of 8-15 mW. We alternated trials between head rotation alone and head rotation combined with LED illumination. The LED was activated for 4 s, centered on the peak velocity of the head rotation.

Histology

For anatomical analysis, mice were transcardially perfused with phosphate buffered saline (PBS) and then with 4% paraformaldehyde (PFA) in PBS. Brains were extracted from the skulls, post-fixed in 4% PFA overnight at 4°C, and subsequently cut with a vibratome to 80-

100 μm thick sequential coronal sections. Slices were collected and mounted in ProLong Gold (Life Technologies) or Vectashield mounting medium containing DAPI (Vector Laboratories H1500). Bright-field and fluorescence images were acquired using an Olympus MVX10 MacroView microscope. For quantifying the number of somata in visual thalamus, deep cerebellar nuclei, and vestibular nuclei (see sections: *Viruses* and *Viral Injections*), neuronal density was counted for each brain slice (visual thalamus: n = 123 slices; deep cerebellar and vestibular nuclei: n = 40 slices) and then averaged. Ipsilateral projections to the injection site, for both DCN and pulvinar tracing experiments were negligible and not considered. The Paxinos brain atlas was used as a reference to delineate these regions.

QUANTIFICATION AND STATISTICAL ANALYSIS

Data Analysis

Unit isolation*:* Automated spike sorting was carried out using KiloSort and KiloSort2 (https://github.com/cortex-lab/Kilosort) by manual curation of the units using Phy and Phy2 (https://github.com/cortex-lab/phy). Single units were identified, and all the following analysis was carried out via MATLAB (MathWorks), but for principal component and decoding analysis (Python 3). The quality of the isolated units was assessed using refractory period violations and stability of amplitude.

Class assignment criteria: To classify the units that are significantly modulated by head movement, we compared its neuronal activity before and during head rotation. The baseline spike rate was calculated on individual trials by averaging the spike rate over a window of 580 or 1000 ms recorded when the platform was stationary before the rotation. The spike rate in response to the rotation of the platform was calculated on the same trials by averaging the spike rate over a window of 580 ms centered around the peak of the rotation velocity profile. Wilcoxon signed-rank tests were then applied to determine if a unit was significantly modulated ($P < 0.05$) by the rotation of the platform. Directional preference of individual unit was quantified by comparing their firing rate in response to CW and CCW rotations and using non-parametric Wilcoxon signed-rank tests, with statistical significance set at $P < 0.05$. This analysis enabled identification of neurons exhibiting preferential responses to specific rotation directions. For each recorded unit, we computed the mean firing rate within a 580 ms temporal window centered on the peak angular velocity of platform rotation. To compare the Z-score of the firing rate collected from 2 populations of mice, we performed Wilcoxon rank sum tests. When reporting averaged absolute Z-score of the firing rate, only neurons significantly modulated by head movement in baseline condition were included. Wilcoxon signed rank tests were performed to compare values obtained in the same recording and only neurons significantly modulated in control condition for a given direction of rotation were considered (i.e., comparing before and during thalamus or contralateral visual cortex silencing).

Cortical depth estimation*:* Cortical depth from pia estimated by using electrophysiological landmarks across layers as described previously (1, 7). Briefly, the Multi-unit (MUA) spectral power (500 Hz to 5 kHz) distribution along the probe track allowed us to locate layer 5a. This approach allowed us to normalize the cortical depth from the pia across mice.

Statistics: Statistical analyses were done using MATLAB and Python 3. No statistical tests were used to predetermine sample size, but our sample sizes are similar to those generally employed in the field. All data are presented as mean ± standard error of the mean (SEM), unless otherwise noted. The stated P values are the results of the non-parametric Wilcoxon rank sum test to compare values between different mice or recordings, and the non-parametric Wilcoxon signed rank test to compare values from the same recording in different experimental conditions. The difference of fraction of neurons modulated across brain areas was accessed using the bootstrap hypothesis test. Specifically, we resampled neurons with replacement from our dataset of Z-score of the firing rate responses to clockwise (CW) and counterclockwise (CCW) rotations (N = 10000 iterations). For each bootstrap sample, we computed response amplitudes and firing rate changes, then fitted Gaussian functions to the cumulative distribution functions to calculate p-values, determining the statistical significance of observed differences. For the anatomical tracing analysis, the non-parametric Wilcoxon rank test was employed to compare the values between slices from the VN and the DCN, as well as between slices from the dLGN and the pulvinar. Experiments and analyses were not blinded.

Cross validated neuron sorting and principal component analysis: For recordings in V1 and pulvinar in control conditions, trials were divided in two equally populated sets (called in what follows training and test sets); a trial averaged response was computed for each neuron separately in the two sets. Neurons were ordered using the UMAP algorithm applied to the training set of the control conditions (8). This ordering was then applied to the test set to obtain the UMAP plots shown throughout the text.
Cross-validated principal component analysis activity (9) was used to estimate the fraction of variance explained by each principal component. In brief, we computed the trial averaged response of neurons in each realization of the training and test sets. The training set was used to derive the principal components, while the test set was used to measure the variance explained in each component. The procedure was repeated ten times with different random realizations of the training and test sets; means and standard errors over realizations are shown in Fig. 1C, 1D, and 2C. As shown by Stringer et al.(9), this method measures the reliable variance of stimulus-related dimensions, excluding trial-to-trial variability from unrelated cognitive and/or behavioural variables or noise. For recordings in V1 and the pulvinar, the first 3 (pulvinar: 5) PCs accounted for 76% and 74% (pulvinar: 87% and 85%) of the variance, respectively.

Relationship between principal components and movement: To quantify the relation between movement and neural activity along each principal component (PC), we defined a predictor y(t) given by

$$y(t) = \sum_{i=1}^{4} \quad a_i \int_{-\infty}^{t} \quad dt' \, e^{(t\prime-t)/\tau_i} x_i(t')/\tau_i,$$

where the index i runs over the movement related variables investigated (speed, velocity, dspeed/dt, acceleration), x_i(t) is the temporal profile of the i-th variable, and each of these variables are convolved with an exponential kernel of amplitude a_i and time constant τ_i . This choice was motivated by the fact that single neuron and population dynamics showed long lasting responses. For each PC, we fitted the corresponding values of a_i and τ_i by minimizing the squared difference between the measured population dynamics and $y(t)$. Analogously with what described for cross validated PCA, fits were performed on a training set, validation was measured on a test set, the procedure was repeated 10 times with random realizations of training and test sets, and the mean and standard error of the predictor performance

(measured with R^2) were evaluated averaging over realizations. Optimization of the temporal profiles for movement-related variables (speed, velocity, dspeed/dt, acceleration) was constrained to $\mathrm{i} < 5$ seconds. This limit prevented large time constants from leading e.g. dspeed/dt to become indistinguishable from speed thus ensuring distinct temporal dynamics. This procedure was effective across PCs, as different kinematic variables, each with distinct temporal structure, captured different aspects of the dynamics. The only exception was PC1, where dspeed/dt contributed similarly to speed due to a fitted time constant near the upper bound, which rendered both temporal profiles very similar to each other. In V1 and the pulvinar, the model captures a large amount of variance in the first 4 PCs (97.2$\pm$0.2, 96.2$\pm$0.7, 93.7$\pm$2.0, and 88.0$\pm$1.3 in V1; and 95.9$\pm$0.9, 93.3$\pm$1.1, 85.5$\pm$4.0, and 80.5$\pm$4.8 in the pulvinar), but then much less in other PCs (55.7$\pm$4.0 in V1 and 18.1$\pm$17.8 in the pulvinar).

Trial to trial variability in the single neuron responses affected our estimates of firing rates and led to different dynamics along each PC in the training and the test sets. These fluctuations are due to the finite number of trials in the experiments; hence they could not be captured by our "kernel" model described above, but can strongly influence our estimate of its performance. To account for this phenomenon in our quantification of the predictor performance, we defined a "null model", which used the dynamics observed in the training set as predictor for the dynamics along each PC in the test set. This null model quantifies the reliability of our estimate of firing rates. It was used as a reference to evaluate the performance of the "kernel" model. Specifically, in each PC we computed the proportion of the variance in the test set explained by the null model (measured with R^2). A value R^2 close to 1 indicates that the estimate of the firing rate of neurons was reliable across training and test sets; a value close to zero or negative, on the other hand, indicates that our estimates of firing rates were mainly determined by trial to trial fluctuations. We found that the null model in V1 and the pulvinar had a positive R^2 only for the first 5 and 6 components, respectively. For PCs with positive R^2, we computed the ratio between R^2 given by the kernel model and the null model; this ratio, which we called the fraction of explainable variance captured, is shown in the bar plots of Figs. 1D and 2C.

To estimate the importance of the i-th variable, we repeated the procedure described above, setting a_j =0 for all $j \neq i$. The analysis in V1 revealed that the first PC was mostly explained by speed and its derivative (Kernel time constants 0.219$\pm$0.004 s and 5.0$\pm$0.1 s); the second PC was mostly explained by velocity and acceleration (kernel time constants 8.56$\pm$0.05 ms and 4.8$\pm$0.1 s); while the third PC was mostly related to speed and its time derivative (kernel time constants 2.62$\pm$0.03 s and 0.674$\pm$0.008 s). Similar results were obtained in the pulvinar.

Decoding analysis*:* Decoding of head movement related information from neural activity was performed by training decoders on spike counts in bins of 100 ms. Decoders were trained on 80% of the bins and tested on the remaining 20%. Figures in the manuscript only show decoder predictions on test bins. To evaluate performance of decoders, training and testing were repeated 100 times, randomly shuffling which trials were used for training and for testing. Decoding performance with shuffled labels were evaluated using the same procedure, but shuffling the association between spikes in a bin and the corresponding animal head movement. Numerical analyses were performed using the python library scikit-learn.

Logistic regression models (solver 'lbfgs' and 'l2' regularization, (10) were used to decode head movement direction. To quantify the dependency of neuronal responses on past head movements, we trained separate decoders for each bin to predict if the corresponding trial was CW or CCW. Training was performed assigning sample weight to each movement value (CW or CCW) that corresponded to its frequency in the dataset. The regularization parameter C of each decoder was determined by maximizing the cross validated performance. Generalization performance across movement profiles was measured using a unique logistic regression model for all the time bins along the trial.

Velocity and acceleration decoding were performed using a nonlinear decoder constructed combining a logistic regression model predicting instantaneous movement direction (three categories: no movement, CW, CCW) and two distinct ridge regression models (corresponding to bins with CW and CCW movements) predicting instantaneous movement magnitude. Unlike those used to characterize the dependency of neuronal responses on past movements, the logistic regression models used here were unique for all the time bins along the trial. The 'l2' regularization parameter of the ridge regression model was optimized to maximize cross validation performance. Training was performed assigning sample weight to each data point; these were computed dividing possible head movement values in 30 equally spaced bins and measuring the frequency of each movement bin in the dataset. We found that this nonlinear decoder outperformed a simpler linear decoder, obtained with a single ridge regression model; this result was likely due to the fact that, unlike the linear decoder, the nonlinear decoder was able to exploit neurons with symmetric CCW and CW response.

To evaluate performance of decoders as a function of the number of neurons, we systematically measured decoding performance as a function of the number of neurons the decoder had access to. For a fixed number of neurons, this was implemented by randomly picking which neurons were used in the decoding and repeating the procedure 1000 times. Mean and standard error of the decoding performance over random realizations of the training and test set are shown in Figs. 1E-G, S2A-C, 2D-F, and S3F,G. In the top-left panel of Figs. 1E, 1F, 1G, S2B, S2C, and S2C, as well as in all of Fig. S2D,F (*SI Appendix*), the individual-colored lines represent independent predictions, each generated from random realizations of the training and test sets.

SUPPORTING INFORMATION: BIBLIOGRAPHY

1. G. Bouvier, Y. Senzai, M. Scanziani, Head Movements Control the Activity of Primary Visual Cortex in a Luminance-Dependent Manner. *Neuron* 108, 500-511.e5 (2020).
2. B. Zingg, *et al.*, AAV-Mediated Anterograde Transsynaptic Tagging: Mapping Corticocollicular Input-Defined Neural Pathways for Defense Behaviors. *Neuron* 93, 33–47 (2017).
3. B. H. Liu, A. D. Huberman, M. Scanziani, Cortico-fugal output from visual cortex promotes plasticity of innate motor behaviour. *Nature* 538, 383–387 (2016).
4. A. D. Lien, M. Scanziani, Tuned thalamic excitation is amplified by visual cortical circuits. *Nat Neurosci* 16, 1315–23 (2013).
5. N. Li, *et al.*, Spatiotemporal constraints on optogenetic inactivation in cortical circuits. *Elife* 8, 1–31 (2019).
6. Z. V. Guo, *et al.*, Flow of cortical activity underlying a tactile decision in mice. *Neuron* 81, 179–194 (2014).
7. Y. Senzai, A. Fernandez-Ruiz, G. Buzsáki, Layer-Specific Physiological Features and Interlaminar Interactions in the Primary Visual Cortex of the Mouse. *Neuron* 101, 500-513.e5 (2019).
8. McInnes L, Healy J, Melville J (2020) UMAP: Uniform Manifold Approximation and Projection for Dimension Reduction. arXiv:1802.03426.
9. C. Stringer, *et al.*, Spontaneous behaviors drive multidimensional, brainwide activity. *Science (1979)* 364 (2019).
10. F. Pedregosa, *et al.*, “Scikit-learn: Machine Learning in Python” Journal of Machine Learning Research 12 (2011) 2825-2830.

SUPPORTING INFORMATION: FIGURE LEGENDS

Figure S1. **V1 neurons respond to head movements with or without compensatory eye movements, related to Figure 1.**
(A) Experimental configuration. Extracellular linear probe in the left V1 of a head-fixed, awake mouse records the response to clockwise (CW) and counterclockwise (CCW) rotations of the table (gray dotted line) surrounded by a virtual drum made of a pattern of vertical light and dark stripes (black dotted line). A camera monitors the right eye through an infrared (IR) mirror. To minimize the number of resetting saccades, we use a lower peak velocity (20 deg/s) as compared to that used in all other experiments (see Methods).
(B) Vestibulo-ocular reflex (VOR) under two conditions: compensation (left) and cancellation (right). Top left: VOR compensation is triggered by table rotations (dotted gray line) in front of a static virtual drum (dotted black line). Bottom left: VOR compensation is characterized by eye movements in the opposite direction but same angular amplitude of the rotating table. Top right: VOR cancellation is triggered by simultaneous rotation of the virtual drum and the rotating table in the same direction. Bottom right: VOR cancellation is characterized by the absence of compensatory eye movements.
(C) Top: UMAP sorting of the average Z-scored FR responses across neurons performing CW (left) and CCW (right) head movements during VOR cancellation. Bottom: Z-scored average of the firing rate for the neurons that are significantly excited (exc.) and suppressed (sup.) by CW (red) and CCW head movement (blue). The gray and black dotted traces on top are the velocity profiles of the table and of the virtual drum, respectively.

Figure S2. Prediction of head movement velocity and acceleration and generalization of head movement information to different head angular rotation profiles, related to Figure 1.
(A) Decoding error of head movement as a function of number of neurons. To obtain these plots, starting from the decoding weight obtained when all neurons are available (e.g. those shown in Figure 1 E-G of the main text and in panels B,C of the current figure), we arrange neurons in decreasing (black) and increasing (red) order of the decoder weight. The errors shown for a given N in the plots are those obtained from a decoder which has access to the first N neurons according to this ordering.
(B) Instantaneous head angular velocity representation: Prediction of head movement velocity (top left), error as a function of number of neurons (top right), decoder weight of single neuron as a function of depth (bottom left), and example neuron selected using highest decoder weight for head angular speed decoding (bottom right). As in Figure 1E-G, light and dark green indicate deep and upper layers, respectively.
(C) As in B but for time derivative of velocity (acceleration).
(D) Prediction of head velocity of a decoder trained on the rising phase (top) and tested on the decaying phase (bottom) of the head movement profile.
(E) Instantaneous head movement direction predicted by a decoder trained on profiles with 80 deg/s peak velocity and tested on other velocity profiles with the same peak velocity but with slower (middle) and faster (bottom) profiles.
Example neurons in panels B,C are marked with purple diamonds. In B,C,E, blue and red traces correspond to quantities measured during CCW and CW rotations, respectively.
(F) Decoding error for single trials in panel E with decoder trained on data (gray) and shuffled control (orange).

Supplementary Figure 3. The pulvinar receives projections from the deep cerebellar nuclei and encodes head angular velocity and acceleration, related to Figure 2.
(A) Experimental strategy. (Top) Injection of Cre dependent Experimental strategy. Top: Injection of Cre dependent transsynaptic anterograde virus (AAV1 hSyn-cre) in the deep cerebellar nuclei (DCN) in a flex-tdTomato reporter line; this approach labels the projections of the DCN and the post-synaptic neurons in red (see Methods). Bottom: Photomicrograph of coronal sections illustrating the injection site in the DCN.
(B) Quantification of the density of neurons receiving projection from the DCN along the anterior posterior axis (A). Comparison of the density of neurons in dorso lateral geniculate nucleus (dLGN, gray) and pulvinar (green) receiving DCN projections.
(C) Experimental strategy. Top: Injection of Cre dependent retrograde virus (rAAV1 hSyn-cre) in the rostro-medial pulvinar in a flex-tdTomato reporter line; this approach labels the neurons that project to the pulvinar. Bottom: Injection site in the rostro-medial pulvinar. Scale bar = 1 mm.
(D) Photomicrograph of coronal sections illustrating retrogradely labelled neurons in the deep cerebellar and vestibular nuclei, respectively. DAPI is in blue and tdTomato is red. Scale bar: 200 µm.
(E) Experimental configuration. Top: Extracellular linear probe spanned the ipsilateral pulvinar in the dorso-ventral axis. Bottom: extracellular linear probe in the pulvinar (green) of a head-fixed, awake mouse records the response to CW and CCW rotations of the table in the dark.
(F) Decoding error of head angular velocity as a function of number of neurons in pulvinar (green) and V1 (black).
(G) As in F but for head angular acceleration.

Supplementary Figure 4. Specificity of muscimol-BODIPY injection in the pulvinar. Photomicrographs of coronal sections showing the distribution of muscimol-BODIPY around the injection site. Top panels: injection site; middle panels: anterior spread; bottom panels: posterior spread. Scale bar: 750 µm. Abbreviations: CL, centrolateral nucleus; DLG, dorsal lateral geniculate nucleus; LH, lateral habenula; LPLR, lateral posterior nucleus, lateral rostral division; LPMR, lateral posterior nucleus, medial rostral division; MDL, mediodorsal nucleus, lateral division; Po, posterior nucleus; VPM, ventral posteromedial nucleus.

Supplementary Figure 5. Decoding error as a function of neural population size across silencing conditions. Each subplot shows direction decoding error probability (y-axis) as a function of the number of neurons, for different silencing conditions during head movements. As in Figure 1E, we used logistic regression models to predict movement direction (CW or CCW) using neural activity in 100 ms bins. Columns represent decoding comparisons: CW vs CCW, Baseline vs CCW, and CW vs Baseline. Rows correspond to different perturbation conditions: (**A**) ipsilateral pulvinar silencing, (**B**) contralateral visual cortex (VC) silencing, and (**C**) combined pulvinar and/or VC silencing. Lines indicate mean error; shaded regions are SEM across bootstrap replicates. Colored bars in insets show the threshold number of neurons required to reach a fixed error level (0.25).

Statistical comparisons quantify the relative increase in the number of neurons required to reach error = 0.25 under different silencing conditions, compared to normal (unsilenced) conditions. Pairwise differences (ΔN and P-values), computed with Welch's t-test on bootstrap samples of neuron thresholds, are as follows. (**A**) **Ipsilateral Pulvinar silencing:** CW vs CCW: ΔN = 392.6%, $P = 6.0\times10^{-6}$; Baseline vs CCW: ΔN = 260.1%, $P = 5.3\times10^{-6}$; CW vs

Baseline: ΔN = 443.4%, P = 1.0×10^{-7}. Silencing has a stronger effect on CW than CCW movements, consistent with the greater response suppression observed during CW movements described in the main text. (**B**) **Contralateral VC silencing:** CW vs. CCW ΔN = -13.3%P = 5.7×10^{-3}; CCW vs Baseline: ΔN = 64.1%, P = 2.2×10^{-3}; CW vs Baseline: ΔN = 37.8%, P = 2.9×10^{-3}. Silencing has a stronger effect on CCW than CW movements, consistent with the greater response suppression observed during CCW movements described in the main text. (**C**) **Combined VC and/or Pulvinar silencing** (here, "ipsi" refers to silencing of Pulvinar on the same side as the recording site, and "contra" refers to silencing of the right visual cortex)**:** CW vs. CCW ΔN = 0.46%, P=0.9; 126.0% (normal vs contra). No bars are shown in the insets for the ipsilateral silencing because the decoding error remained above 0.25 across all population sizes tested, preventing reliable estimation of the threshold number of neurons. Baseline vs CCW: ΔN = 126.0%, P = 2.2×10^{-4} (normal → contra); ΔN = 293.5%, P = 7.2×10^{-9} (normal → ipsi); ΔN = 380.4%, P = 1.7×10^{-8} (normal → ipsi+contra); ΔN = 74.1%, P = 1.2×10^{-4} (contra → ipsi); ΔN = 112.6%, P = 1.0×10^{-6} (contra → ipsi+contra); ΔN = 22.1%, P = 5.4×10^{-4} (ipsi → ipsi+contra). CW vs Baseline: ΔN = 7.1%, P = 0.36 (normal → contra); ΔN = 256.3%, P = 6.2×10^{-7} (normal → ipsi); ΔN = 445.0%, P = 9.3×10^{-6} (normal → ipsi+contra); ΔN = 232.6%, P = 8.4×10^{-7} (contra → ipsi); ΔN = 408.9%, P = 1.0×10^{-5} (contra → ipsi+contra); ΔN = 53.0%, P = 1.6×10^{-4} (ipsi → ipsi+contra). Silencing the right visual cortex following pharmacological inactivation of the left pulvinar led to a further reduction in decoding performance (i.e., larger ΔN) beyond that caused by pulvinar silencing alone, consistent with the hypothesis that the ipsilateral pulvinar and contralateral cortex independently contribute to head movement responses in V1.

Figure S1

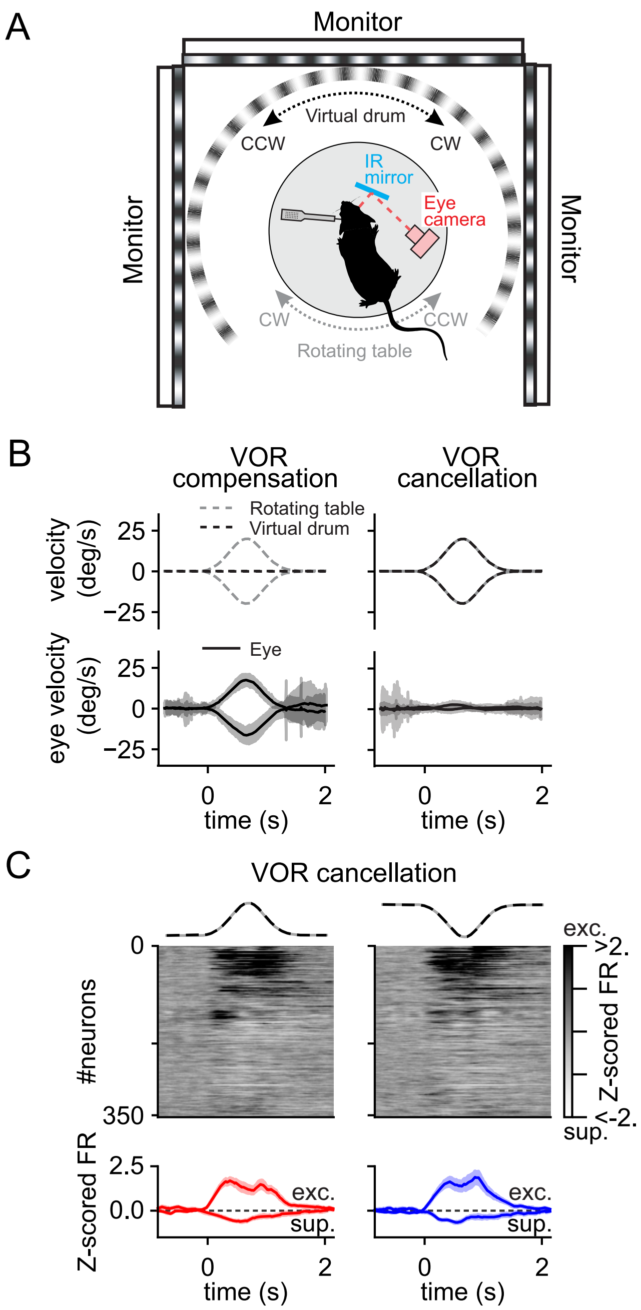
A
Monitor
Virtual drum
CCW
CW
IR
mirror
Eye
camera
Monitor
Monitor
CW
CCW
Rotating table
B
VOR
compensation
VOR
cancellation
Rotating table
Virtual drum
velocity
(deg/s)
25
0
−25
Eye
eye velocity
(deg/s)
0
2
time (s)
C
VOR cancellation
0
350
#neurons
exc.
>2.
Z-scored FR
<-2.
sup.
Z-scored FR
2.5
0.0
exc.
sup.
time (s)

Figure S2

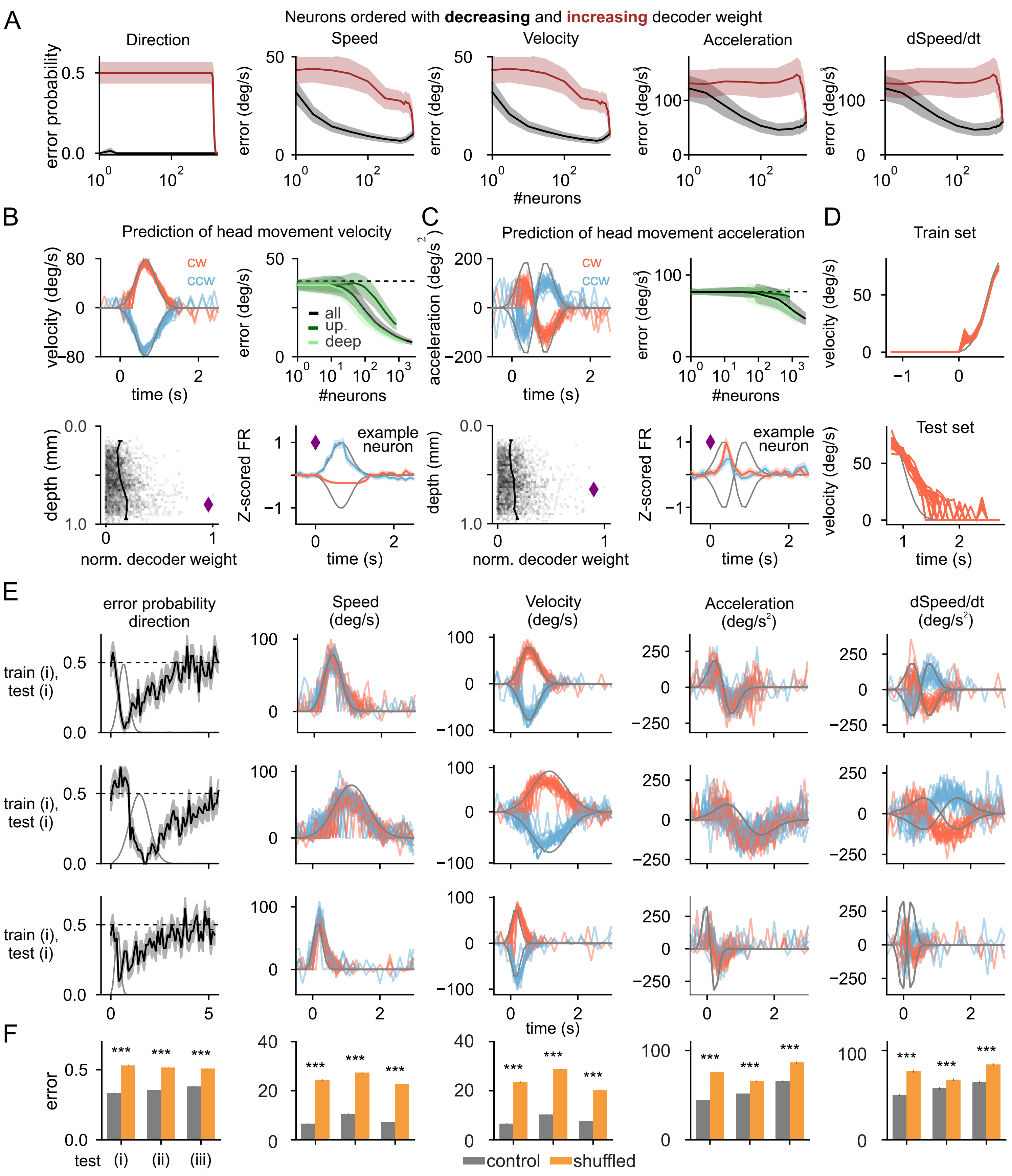

A
Neurons ordered with decreasing and increasing decoder weight
Direction
Speed
Velocity
Acceleration
dSpeed/dt
error probability
error (deg/s)
error (deg/s³)
#neurons
B
Prediction of head movement velocity
velocity (deg/s)
cw
ccw
time (s)
all
up.
deep
#neurons
depth (mm)
norm. decoder weight
Z-scored FR
example neuron
C
Prediction of head movement acceleration
acceleration (deg/s²)
D
Train set
Test set
velocity (deg/s)
E
error probability direction
Speed (deg/s)
Velocity (deg/s)
Acceleration (deg/s²)
dSpeed/dt (deg/s³)
train (i), test (i)
time (s)
F
error
test (i) (ii) (iii)
***
control
shuffled

Figure S3

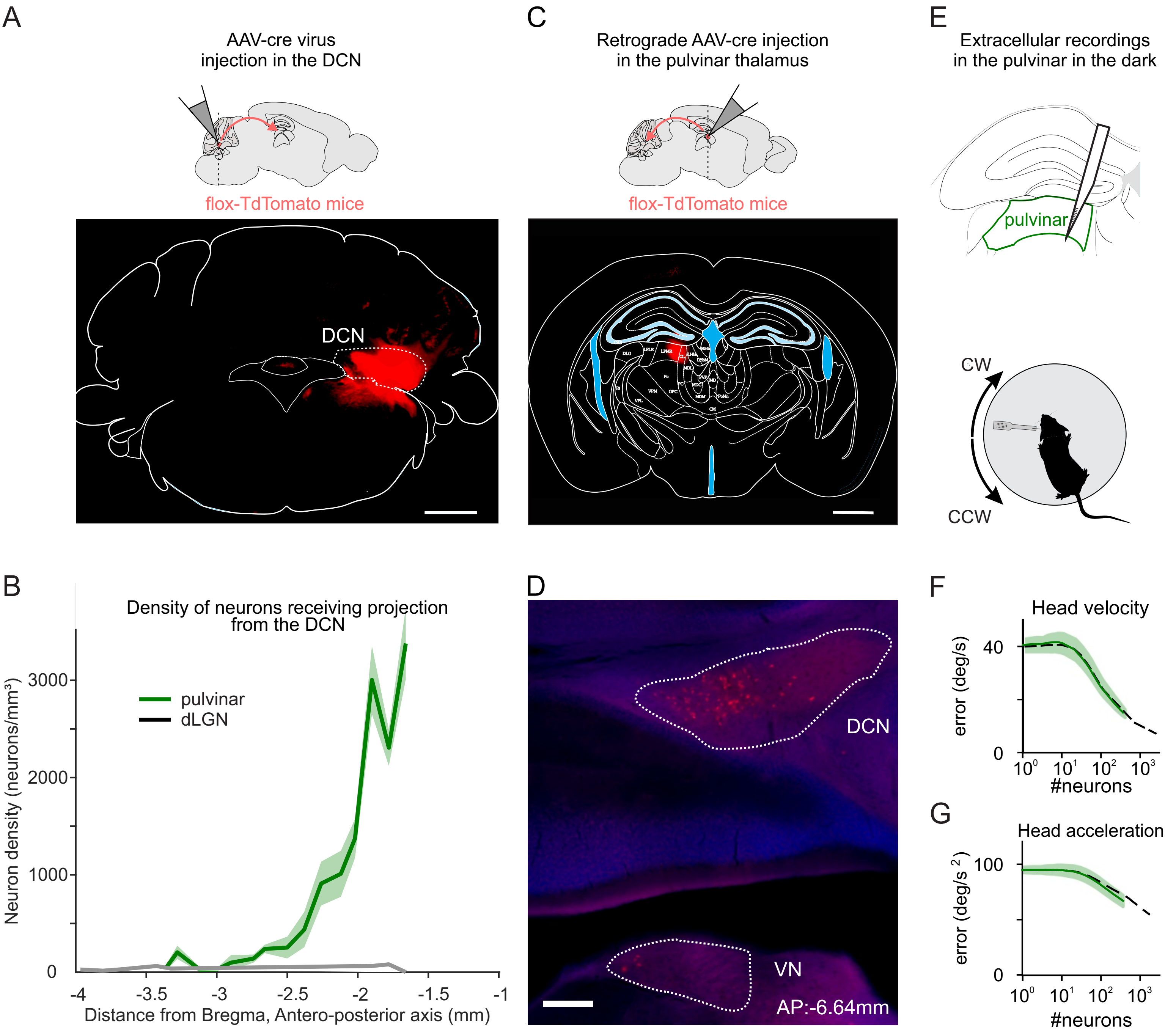
A
AAV-cre virus injection in the DCN
flox-TdTomato mice
DCN
B
Density of neurons receiving projection from the DCN
pulvinar
dLGN
Neuron density (neurons/mm³)
3000
2000
1000
0
-4
-3.5
-3
-2.5
-2
-1.5
-1
Distance from Bregma, Antero-posterior axis (mm)
C
Retrograde AAV-cre injection in the pulvinar thalamus
flox-TdTomato mice
D
DCN
VN
AP:-6.64mm
E
Extracellular recordings in the pulvinar in the dark
pulvinar
CW
CCW
F
Head velocity
error (deg/s)
40
0
10⁰
10¹
10²
10³
#neurons
G
Head acceleration
error (deg/s ²)
100
0
10⁰
10¹
10²
10³
#neurons

Figure S4

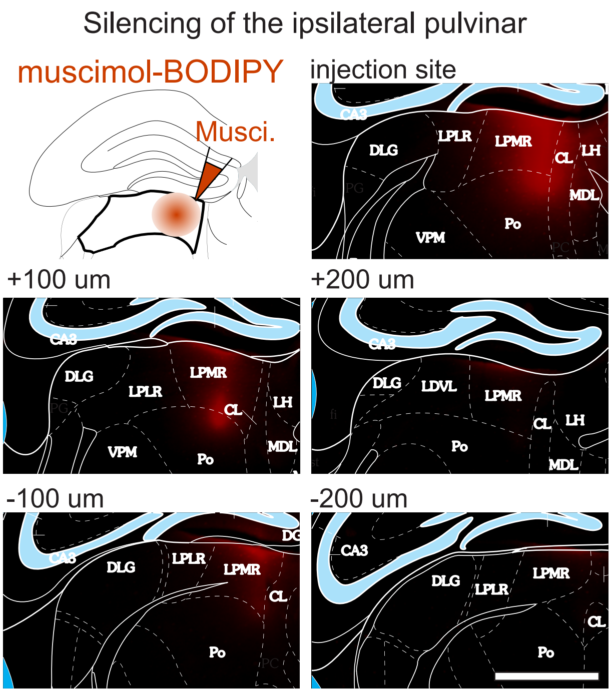
Silencing of the ipsilateral pulvinar
muscimol-BODIPY
Musci.
injection site
CA3
DLG
LPLR
LPMR
CL
LH
MDL
Po
VPM
+100 um
CA3
DLG
LPLR
LPMR
CL
LH
VPM
Po
MDL
+200 um
CA3
DLG
LDVL
LPMR
CL
LH
Po
MDL
-100 um
CA3
DLG
LPLR
LPMR
DG
CL
Po
-200 um
CA3
DLG
LPLR
LPMR
CL
Po

Figure S5

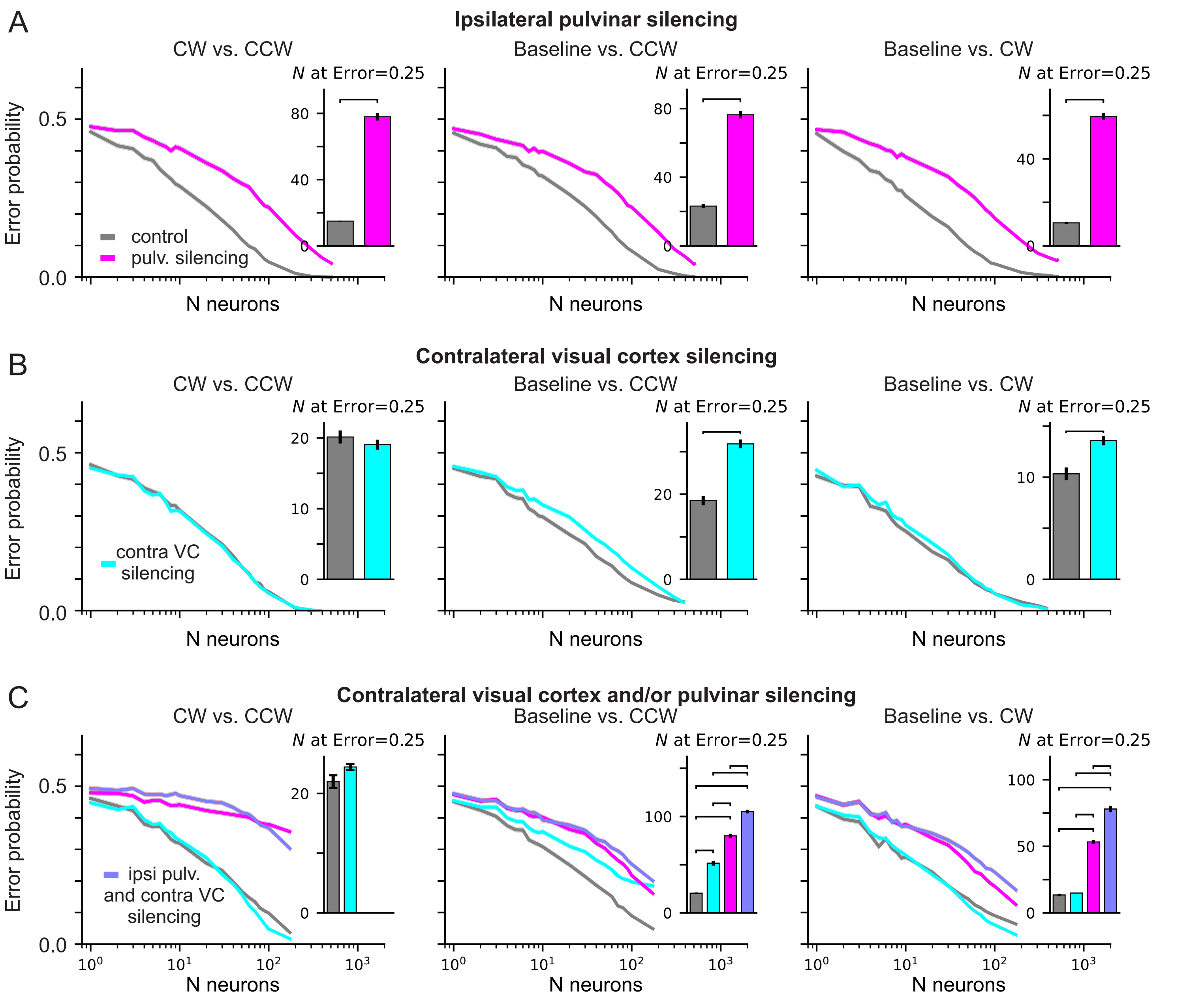
A
Ipsilateral pulvinar silencing
CW vs. CCW
Baseline vs. CCW
Baseline vs. CW
N at Error=0.25
Error probability
N neurons
control
pulv. silencing
B
Contralateral visual cortex silencing
contra VC silencing
C
Contralateral visual cortex and/or pulvinar silencing
ipsi pulv. and contra VC silencing
